# Genetic Delivery and Gene Therapy in Pulmonary Hypertension

**DOI:** 10.3390/ijms22031179

**Published:** 2021-01-25

**Authors:** Nabham Rai, Mazen Shihan, Werner Seeger, Ralph T. Schermuly, Tatyana Novoyatleva

**Affiliations:** 1 Excellence Cluster Cardio-Pulmonary Institute (CPI), Universities of Giessen and Marburg Lung Center (UGMLC), Member of the German Center for Lung Research (DZL), Justus Liebig University of Giessen, Aulweg 130, 35392 Giessen, Germany; nabham.rai@innere.med.uni-giessen.de (N.R.); mazen.shihan@innere.med.uni-giessen.de (M.S.); werner.seeger@innere.med.uni-giessen.de (W.S.); ralph.schermuly@innere.med.uni-giessen.de (R.T.S.); 2 Max Planck Institute for Heart and Lung Research, 61231 Bad Nauheim, Germany; 3 Institute for Lung Health (ILH), 35392 Giessen, Germany

**Keywords:** pulmonary hypertension, gene therapy, viral gene delivery

## Abstract

Pulmonary hypertension (PH) is a progressive complex fatal disease of multiple etiologies. Hyperproliferation and resistance to apoptosis of vascular cells of intimal, medial, and adventitial layers of pulmonary vessels trigger excessive pulmonary vascular remodeling and vasoconstriction in the course of pulmonary arterial hypertension (PAH), a subgroup of PH. Multiple gene mutation/s or dysregulated gene expression contribute to the pathogenesis of PAH by endorsing the proliferation and promoting the resistance to apoptosis of pulmonary vascular cells. Given the vital role of these cells in PAH progression, the development of safe and efficient-gene therapeutic approaches that lead to restoration or down-regulation of gene expression, generally involved in the etiology of the disease is the need of the hour. Currently, none of the FDA-approved drugs provides a cure against PH, hence innovative tools may offer a novel treatment paradigm for this progressive and lethal disorder by silencing pathological genes, expressing therapeutic proteins, or through gene-editing applications. Here, we review the effectiveness and limitations of the presently available gene therapy approaches for PH. We provide a brief survey of commonly existing and currently applicable gene transfer methods for pulmonary vascular cells in vitro and describe some more recent developments for gene delivery existing in the field of PH in vivo.

## 1. Introduction 

Cardiovascular diseases (CVDs) are the number one cause of death worldwide [1]. CVDs represent a group of various disorders affecting the blood circulatory system and the functionality of the heart and blood vessels [2]. Despite the diversity of underlying mechanisms, plentiful CVDs are characterized by narrowing or obstruction of blood vessels that ultimately outcome in various pathological settings, as acute myocardial infarction, chest pain (Angina pectoris), stroke, and pulmonary hypertension (PH) [3,4]. PH comprises a group of pulmonary vascular disorders, including pulmonary arterial hypertension (PAH) [5]. PAH is a complex disease of multifactorial pathobiology, in which the loss and remodeling of pulmonary blood vessels, ultimately results in progressive functional decline and right heart failure [6]. Remodeling of the pulmonary vessel wall is tightly associated with enhanced proliferation and dysregulated apoptosis of pulmonary vascular cells on one site and perivascular infiltration of inflammatory cells on another [5,7]. Vasoconstriction of remodeled vessels and thrombosis contribute to hemodynamic alterations of the pulmonary circulation and an increase of pulmonary vascular resistance in PAH [8,9]. The enhanced pulmonary vascular resistance increases shear stress-induced pulmonary vascular endothelial injury/dysfunction, and together with the vessel loss represent major hallmarks of the pathogenesis of PAH [5,8]. Pulmonary circulation represents a unique low-pressure, high-flow system receiving all cardiac output with its primary aim of respiratory gas exchange [10]. The pulmonary vascular wall consists of three layers, including adventitial fibroblast, medial smooth muscle, and intimal endothelial cell (EC), all of which actively contribute to the pathogenesis of PAH. All pulmonary vascular constituents are involved in the complex process of vascular remodeling and formation of the pathological blood vessel [11]. Concomitantly to the cellular heterogeneity of each vascular compartment, the contribution of other cells to PAH, as inflammatory cells and platelets is well recognized.

Recent progress in genetic approaches allowed discovering genes and genetic loci responsible for predisposition or emergence of PH [12]. Amongst those genes is the bone morphogenetic protein receptor type 2 gene (*BMPR2*), a member of the transforming growth factor-β (TGF‑β) superfamily, which mutations are recognized in around 70–80% of families with PAH and 10–20% of idiopathic PAH [13,14,15]. Moreover, the mutations in genes encoding various factors associated with BMP receptor signaling such as *SMAD1*, *SMAD4*, *SMAD9*, activin receptor-like kinase 1 (*ACVRL1*), and endoglin (*ENG*), were also identified in PAH [16]. Mutations in *CAV1*, which encodes caveolin‑1 [17], *KCNK3* (potassium channel subfamily K member 3) [18], *TBX4* (T‑box 4) [19,20], *ATP13A3* (ATPase 13A3), *AQP1* (aquaporin 1), *SOX17* (SRY‑box 17), and *GDF2* (growth differentiation factor 2/BMP9) [21] have been also discovered in PAH. As various defective genes and malfunctioned gene expression is involved in the predisposition and the emergence of PAH/PH, the development of safe and efficient gene‑delivery systems represents a promising therapeutical approach for the treatment of disease. Though medications approved for use in treating PAH by the Food and Drug Administration (FDA), in general, improve hemodynamics parameters and quality of life of patients with PAH, no current PAH therapy satisfies all of the requirements, as they do not correct the fundamental mechanistic insights underlying the disease occurrence [22]. Furthermore, while regenerative cell therapy approaches may represent an effective therapy in general, the results of clinical trials employing cell-therapy approaches provided relatively modest beneficial outcomes, in comparison to pre‑clinical experimental reports [23]. Based on this knowledge and considering recent great advances in gene‑editing technologies, the gene therapy approaches that would correct disturbed/defective gene expression profile within the lung vasculature might be a realistic future option. The present review will gain insight into various gene delivery strategies, the delivery of specific genes of interest, and target cells, mainly pulmonary vascular cells, in the context of PH.

### 1.1. Pulmonary Endothelium in Pulmonary Hypertension

The endothelium represents a semi‑permeable interface between hemodynamics and the underlying vascular wall [24]. The pulmonary endothelium lining of the normal lung is characterized by significant heterogeneity, by both functional diversity of endothelium along the arterial, capillary, and venous side of the circulation, as well as heterogeneity within cell population [25,26]. The main tasks of the healthy pulmonary endothelium are maintenance of homeostasis, vascular tone, reactivity and growth, regulation of blood flow, control of thrombosis and thrombolysis, platelet and leukocyte trafficking, production of molecules with autocrine and paracrine effects, and physical barrier function [25,27,28]. Dysfunction of these endothelium-dependent regulatory systems caused by vascular injury mediated by shear stress, viral infection, and alveolar hypoxia converts healthy endothelium to the pathological endothelium [25]. Fragmentation of the internal elastic lamina and in situ thrombosis are prominent features in patients with various vascular diseases. EC injury and dysfunction is one of the first steps to occur in PAH [29]. Pulmonary endothelial dysfunction is characterized by the shift of endothelial-dependent vasodilatation toward vasoconstriction. Vascular injury disturbs EC-maintained homeostasis by altering permeability, by producing coagulating [30], vasoconstrictive and pro-proliferative factors [28], and by reducing the production of vasodilatory and anti-proliferative mediators [31]. Thus, endothelium serves as an attractive target for the gene therapy approach because of its great accessibility and significance in the pathophysiology of PAH. Cultured primary pulmonary ECs represent an excellent model system to study many aspects of various pulmonary diseases, including PH, in which both non‑viral and viral-mediated approaches are established.

### 1.2. Smooth Muscle Layer in Pulmonary Hypertension

During pulmonary vascular dysfunction, the smooth muscle cell (SMC) layer undergoes various transformations, including hypertrophy, hyperplasia, and matrix protein deposition [32]. Hypertrophy plays a major role in larger‑sized more proximal vessels, whereas hyperplasia is more prevalent in the smaller resistance arteries [33]. The shift from contractile to synthetic phenotype occurs in SMCs of hypertrophied arteries [34]. Proximal versus peripheral arteries show SMCs heterogeneity leading to different signaling pathways in distinct areas of the lung as evident by using antibodies directed against contractile proteins [35]. The proliferative status and anti-proliferative properties of SMCs vary to a wider degree depending on their anatomical localization [36]. In hypertension pro-hypertensive impetus, including mitochondrial oxidative stress, mechanical forces, and hemodynamic changes, results in vascular structural, functional, and mechanical changes through stimulating pathological signaling pathways [37,38]. Aberrant changes in SMC proliferation and metabolic reprogramming are some of the primary components in PAH pathophysiology. These metabolic disturbances not only increase pulmonary artery (PASMC) proliferation but also diminish apoptosis, leading to an aberrant pulmonary vascular remodeling [39,40]. In PAH, a metabolic shift to glucose metabolism is due in part to alterations of a pyruvate kinase M2 (PKM2)/PKM1 ratio [41,42]. Furthermore, a decay in PKM2 tetramers has been described in PAH-PASMCs [43], while PKM2 activation, possibly by promoting tetramer formation, protected against the proliferation and migration of PASMCs [44]. Another enzyme, targeted in PH is pyruvate dehydrogenase (PDH). The inhibition of PDH, by Hypoxia-inducible factor-1α (HIF‑1α) dependent pyruvate dehydrogenase kinase (PDK), uncouples glycolysis from glucose oxidation ultimately promoting PASMC proliferation [45,46]. Together, various metabolic alterations, denoted by excessively elevated glycolysis comparatively with pyruvate utilization and glucose oxidation enhance cell proliferation and impair apoptosis [47,48]. Amongst other factors responsible for this uncontrolled proliferative status in PAH, is the deficiency of the Bone Morphogenetic Protein Receptor Type 2 (BMPR2) signaling pathway, which represents an essential characteristic of PASMCs from patients with PAH [49]. Furthermore, an upregulated expression and/or activation of receptor tyrosine kinases (RTKs) and their corresponding effectors [50,51,52], combined with deregulated activity of CDKs-cyclin complexes [53] cumulatively trigger the changes of various transcription factors, such as HIF‑1 [54], Forkhead box O (FOXO) [55], Nuclear Factor of Activated T-cells (NFAT) [56]. The modulation from contractile to synthetic SMC phenotype produces a high level of vasoconstriction in PAH and many signaling pathways are implicated in the regulation of contractility [57]. Similarly to primary ECs, gene delivery to primary SMCs can be accomplished by using viral or non-viral vectors.

### 1.3. Pulmonary Arterial Adventitia in Pulmonary Hypertension

Another important regulator of pathophysiological remodeling of pulmonary vessels is the adventitial fibroblast (AF). As opposed to the principle cellular components of intima and media, i.e., ECs and SMCs, AF attracted attention to its study much later [41,58,59,60]. As evident by recent reports, AFs critically contribute to the development of various CVDs [61,62,63]. Early and remarkable remodeling of the adventitial layer has been recognized in various experimental models of systemic vascular injury and PH [64,65]. Originally, AFs were believed purely to support the framework providing mechanical stability by producing the extracellular matrix (ECM) proteins. Nowadays, AF is recognized as a critical regulator of vascular wall function [58]. AFs together with the other types of cells of the adventitia suggested to “sense” the hypertensive state of the injured vessel and respond by activation [58,66]. Activated AFs are reacted by enhanced proliferation and differentiation, production of ECM proteins and adhesion molecules, and release of cytokines, chemokines, and growth factors [58,64]. Collagen deposition is one of the major determinants for anti-vasodilatory effects in remodeled vessels where pulmonary AFs deposit Type 1 Collagen around the vessels, which along with elastin synthesis may lead to stenosis [67]. All of these events cumulatively affect smooth muscle layer function and pulmonary circulation leading to PH [41,68,69]. The interplay among mentioned above pulmonary vascular cells, and various other players, as pericytes, platelets, immune, and inflammatory cells contribute to pathophysiological features of PAH.

## 2. Gene‑Delivery Approaches and Gene Therapy

Current conventional pharmacological treatments on intracellular proteins render itself less effective, as genes encoding these targets are unaffected. Mutations or abnormal regulations of gene expression alter the cellular phenotype and contribute to PH pathogenesis by elevating proliferative characteristics, subduing apoptotic resistance, and modifying calcium equilibrium [70]. Multiple lines of evidence indicate that mutations of numerous genes are etiologically associated with the development of PH [14,19,24]. Therefore, gene-therapy holds promise, as a new treatment to provide a clinically therapeutic effect. In vitro gene delivery to primary vascular cells can be employed both for vascular tissue engineering and regeneration [71], and for studying gene function under normal homeostasis and pathophysiological conditions [72]. The transfer of biomacromolecules, such as nucleic acids (NAs) across the cellular membrane into the cellular cytosol is a critically essential and fundamental step for successful gene therapy. The introduction of exogenous genetic material into vascular cells shows great promise for the treatment of PAH. While the challenge of transfection is adequately addressed for many immortalized cell types in vitro, transfection of primary vascular cells remains a significant struggle. Moreover, even when high transfection efficiencies are achieved, toxic and off-target effects may provide confound results. Thus, while there has been a huge amount of work on refining transfection approaches over the last decades, unresolved frontiers still exist. The intrinsic problem of introducing negatively charged biomolecules such as DNA and RNA into the cells with a negatively charged membrane overcomes with transfection, which can be chemical, physical, or biological.

### 2.1. Non‑Viral Delivery Dystems

#### 2.1.1. Chemical Transfection

Classical methods of chemical transfection majorly include polymers (polyplex), cationic liposomes (lipoplex), the combination of both (lipopolyplex), and the calcium phosphate (CaP) [73]. In almost all of these chemical techniques, the transfection reagents are complexed with the DNA to neutralize its negative charge, to condense the DNA, mediating interaction with the cell membrane, and promote entry into the cell [73]. Despite the vast majority of different studies demonstrating effective transfection of small interfering RNAs (siRNAs) in primary pulmonary vascular cells [74,75], research on the transfer of various transgenes by non-viral approaches in primary pulmonary vascular cells is limited [75,76,77,78]. The major hindrance to the transfection of primary vascular cells by non-viral approaches is the low efficiency of gene transfer, as the results obtained from surrogate model cell lines do not reflect the situation in primary cells [79]. Furthermore, a limited lifespan in vitro and a great interindividual variety of primary lung vascular cells hinder their applications in functional genomics and gene function analysis. The era of efficient transfection of cultured mammalian cells initiated with the discovery and the implementation of synthetic cationic polymers as polysaccharide diethylaminoethylene (DEAE)‑dextran, polybrene, polyethyleneimine (PEI), and dendrimers [80,81,82,83]. Polymers, by coating and neutralizing negatively, charged DNA, form nanoscale polymer-DNA complexes (polyplexes). PEI, one of the earliest developed lipid-based cationic polymers, is characterized by its strong DNA compaction capacity. PEI is proven to destabilize lysosomal membranes subsequently by enabling PEI-NA complexes to evade degradation in lysosomal vesicles, which ultimately results in high transgene expression [84]. PEI-based transfection reagents have shown improved transfection efficiency albeit with some cytotoxicity depending on the cell type and type and size of cargo (siRNA, ribozymes, oligonucleotides, plasmid DNA) [85,86]. PEIs have been used as a non-viral gene delivery system in vascular cells extensively [87,88,89,90,91,92]. Various architectures and formulations of PEI strongly impact the physical-chemical properties of PEI/NA complexes and transfection effectiveness in vitro [86]. PEI is very efficient in the delivery of plasmid DNA and siRNAs in vivo after lung instillation [93], by aerosol delivery, and systemically [94,95]. Interestingly, aerosolization of PEI was reported to be more proficient than the intratracheal intubation of the lungs [96]. PEI/NA complexes delivered intravenously in 5% glucose into adult mice resulted in very high luciferase expression in ECs of small vessels and pneumocytes [97]. PEI mediated gene delivery in vivo is regulated by various physicochemical parameters, such as molecular weight, structure (branched or linear), PEI/NA ratios, and purity of the polymer. Furthermore, organ distribution and gene expression are largely dependent on the route of administration or the type of PEI used [86]. Unfortunately, poor persistence of PEI-mediated gene expression, prominent cellular toxicity, pro-inflammatory properties, and severe systemic side effects limit the use of PEI polyplexes for gene transfer in vivo [86].

Another approach, known as calcium phosphate (CaP) co‑precipitation suitable for transfection of a vast variety of mammalian cells, was developed later on [98]. CaP gene transfer is an easy to use, cost-effective, and safe method of gene transfer. CaP-mediated co-precipitation has been demonstrated to augment lentiviral transduction of cardiac myocytes and vascular SMCs, enhancing both the efficiency and the speed of gene transfer, offering an improved tool for potential gene therapy applications [99]. Insufficient transfection efficiency, tightly regulated by very strict physicochemical conditions of the reaction mixture, and poor reproducibility represent critical disadvantages of this specific technique [100]. To overcome these obstacles, CaP‑nanoparticles became a promising tool for transfection due to their high biocompatibility and easy biodegradability. As an example, the DNA-functionalized CaP‑nanoparticle-loaded collagen scaffolds implanted in rats demonstrated successful BMP-2 release in vivo [101]. Despite that CaP nanoparticles provide the benefits of low toxicity, and high DNA entrapment efficiency, their in vivo transfection efficiency, remained up to now low [102]. The invention of lipid-based cationic polymers in which the cationic group of the lipid links with negatively charged phosphates on the NA, for an easy transfer of exogenous genetic material across the plasma membrane, was a breakthrough step in gene delivery progress [98]. Liposome-mediated delivery offers multiple advantages over chemical reagent-mediated transfection, such as higher efficiency of gene transfer, the ability to transfect a broader number of cells, an effective delivery of genetic material, and in vitro and in vivo applications [103,104]. The transcripts can be read as fast as within some hours to 4–5 days [105]. To improve transfection efficiency and lower cytotoxicity, non-viral gene delivery solutions are constantly altered and modified. Lipofectamine comes under the category of lipid-based cations which form liposomes by taking up the cargo and give a positive charge to the complex of NA: liposomes. The liposomal complex merges easily with the cell membrane as both of them are comprised of the phospholipidic bilayer. The complex then enters the cells via endocytosis and is delivered into the cytosol for further cellular action [98]. A wide variety of Lipofectamines are commercially available nowadays (Lipofectamine 2000, 3000, RNAiMAX). Depending on the cell type all of them exhibit various efficacy of transfection and toxicity [106]. Nowadays, Lipofectamines remained the standard transfection choice and extensively utilized as a non-viral gene delivery system in primary cells in vitro [107,108,109]. In the context of PAH the Lipofectamines are broadly utilized in all three primary pulmonary vascular cells in vitro [75,110,111,112].

An alternative to liposomal transfection reagents is non-liposomal polymers that are capable to form complexes with DNA or RNA, and the other types of lipids by forming micelles in aqueous solutions. FuGENE^®^ 6 and FuGENE^®^ HD transfection reagents exhibit low toxicity and high efficiency in a variety of cell types and are suitable for cells that are hard to transfect. Transient transfection using FuGENE non‑liposomal reagents was reported for human pulmonary arterial ECs (PAECs) [113] and bovine PASMCs [114]. In vitro, various factors influence the efficiency of the delivery system such as the size of genetic cargo, nuclei acid to reagent ratio and the cell surface entry, subsequent dissociation of NA/reagent complex, and NA transport into the nucleus [115]. The application of nanoparticles (NPs), a novel class of drug delivery systems used for the transport of drugs to target organs, has opened new horizons in the treatment of CVDs, and particularly PAH. Treatment with established vasodilators such as prostacyclin (PGI_2_), Endothelin Receptor Antagonists (ERAs), Phosphodiesterase Type 5 (PDE5) Inhibitors, and Soluble Guanylate Cyclase (sGC) Stimulator‑loaded NPs have been reported to be effective both in vitro and in experimental PAH models in vivo [116]. Recently, gene therapies utilizing NPs have been also tested for PAH. Intratracheal instillation of transcription factor nuclear factor κB (NF‑κB) decoy oligonucleotide-bioabsorbable polymeric NPs formulated from a poly‑(ethylene glycol)‑block‑lactide/glycolide copolymer (PEG–PLGA) attenuated the development of monocrotaline (MCT)‑induced PAH [117]. An intravenous liposomal NP delivery of an antisense oligonucleotide against microRNA‑145 was reported to improve Sugen Hypoxia-induced PAH in rats [118].

#### 2.1.2. Microinjection

Direct microinjection into primary cells or nuclei is effective although laborious technique to deliver genetic material into the cells using a fine needle [119,120]. Microinjection technology is a widely used method to effectively transfer oligonucleotides, siRNA and short hairpin RNA (shRNA) vectors into a single cell, which has been implemented and improved over the last century to achieve accurate and precise transfection. Pronuclear injection and genomic editing/targeting result in successful genetic manipulation of mammalian blastocytes and the generation of transgenic mice [121,122,123,124].

In addition to being used for delivering various NA, microinjection is utilized to transfer peptides, proteins, antibodies, hormones, and various drugs into single cells below the range of cytotoxicity with reliable results and readouts [125,126,127,128,129]. The pronuclear microinjection of NA constructs into the one-cell stage zygote remains the most commonly used technique to generate transgenic mice [130]. With the invention of the clustered regularly interspaced short palindromic repeats/CRISPR-associated 9 (CRISPR/Cas9) system technology for gene targeting, pronuclear injection gained additional importance [131]. Many transgenic mice generated by pro-nucleus microinjection for gene targeting purposes were employed in the context of PH. Microinjection of mRNA that encodes the zinc-finger nucleases that recognize the *BMPR2* sequence resulted in the generation of the first genetically modified rat model linked to *BMPR2* mutations (BMPR2(Δ140Ex1/+) rats). Importantly, the deficiency of BMPR2 signaling is tightly associated with endothelial to mesenchymal transition (EndoMT) and is involved in the occlusive vascular remodeling of PAH [132]. Another example of transgenic mice used to investigate PH is the SMC‑specific Rho‑associated coiled-coil containing protein kinase 2 (ROCK2)‑deficient mice, which were created by microinjection of *SM22α/ROCK2* DNA fragment into the mouse oocyte [133]. The matrix metalloproteinase‑9 (MMP‑9) transgenic mice, generated by microinjection of human MMP-9 cDNA construct into fertilized mouse oocytes, demonstrate exacerbated PH upon serial administration of MCT [134]. Microinjection of human pulmonary vascular SMCs with dominant-negative phosphatidylinositol 3-kinase (PI3K) class IA inhibited platelet-derived growth factor (PDGF)‑induced synthesis of DNA by 65%, resulting in altered proliferation and migration of human pulmonary vascular SMCs [135]. Other researchers investigated PH resulting from occlusive lung arterial lesions in pulmonary ECs by implementing microinjection to insert the Fas-induced apoptosis (FIA) construct under Tie‑2 promoter into ova from superovulated CD1 mice [136]. Human PAECs were microinjected with transient receptor potential‑1 (Trp1) specific-mRNA‑antisense to inhibit the store‑operated calcium [Ca^2+^] entry response/pathway to inflammation [137]. Microinjection of Angiotensin II (Ang II) in vascular SMCs proven to cause a rapid increase in [Ca^2+^] in the cytosol and the nucleus [119,138]. Although relatively inefficient, microinjection is still used to deliver the cargo directly into the cells using nanocarriers due to its low toxicity [139]. Nanocarriers can bypass the cellular processes and can remain in the cells for several days. Altogether, microinjection comes with its challenges of decreased cell viability, changed morphology, and loss of transgene [140].

#### 2.1.3. Electroporation

Electroporation is another physical method of gene transfer with the use of an electrical pulse to disturb the plasma membrane, forming pores consequently allowing the passage of NA in the cells [93]. In contrast to the viral‑based techniques, electroporation is applied to transport a huge range of various molecules in a variety of cell types in vitro [73,94]. The technique requires optimization of pulse duration and strength for each cell type and has been efficiently optimized for the transfer of a neo-resistance carrying plasmid in vascular ECs [95]. Efficient and safe delivery of RNAs in ECs is rarely achieved and electroporation as a successful delivery system is evident by the transfection of exosome/siRNA complex into the pulmonary microvascular ECs against intercellular adhesion molecule‑1 (ICAM‑1), found to play an important role in acute lung injury (ALI) [96]. In numerous CVDs, vascular SMCs respond to the stimuli by changing their plasticity in the phenotype and switch from a quiescent contractile to a more pro‑proliferative one [97]. The inhibition of SMC proliferation and subsequent reduction in intimal hyperplasia was accomplished by electroporation of the monocyte chemoattractant protein‑1 (MCP‑1) [141]. The nucleofector-employed transfection of human PASMCs and human pulmonary arterial adventitial fibroblasts (PAAFs) with plasmid encoding of Ras association domain family 1A (*RASSF1A*) gene, a key factor of hypoxic signaling in PH, enhanced proliferative response of both cell types [75]. In normal human lung fibroblasts, targeting leptin via electroporation linked to inhibiting inflammatory mediators and thus improving airway remodeling in asthma [99]. Non‑viral gene transfer of miR‑145 by electroporation in SMCs demonstrated to prevent vein graft disease by regulating SMC plasticity [100]. The usage of pulse on the plasma membrane is a harsh method often resulting in substantial cell death thus extensive optimization of the technique is often required [103]. Electroporation in vitro comes with its limitations such as inefficiency, cytotoxicity, low cell viability, and inflammatory response [142,143,144]. To overcome the aforementioned obstacles, researchers optimized electroporation ex vivo and in vivo approaches in the lung [143,144]. The advantages and the limitations of gene transfer into the pulmonary vascular tissues via electroporation are described by various reports conducted over the last twenty years [103,143,145]. Gene therapy and electroporation mediated‑gene transfer in vitro and ex vivo still do not fully recapitulate the complex process of in vivo electroporation. The mechanical and shear stress forces, the contribution of the immune system, action of hormones might cause the alterations of cellular responses and signaling pathways ultimately affecting successful gene delivery [103,146,147,148,149]. Cyclic stretching of the murine lungs by ventilation and transthoracic electroporation of DNA plasmid leads to increased transfection efficacy up to 4‑fold over murine lungs without ventilation [150]. One would expect that pulsatile blood flow, tension, and shear stress forces play a major role in the electroporation efficiency of the pulmonary vascular system. The first invention of electroporation in vivo approaches was thirty years back. Electroporation was efficiently utilized in the lungs in vivo [142,144]. Successful electroporation to overexpress specific genes, treat diseases such as cancer [151], or even for vaccination purposes [152] is reported. Lung electroporation was performed through the delivery of NAs through the airways (intratracheal aerosolization) by aspiration/inhalation and applying electric pulses on the chest of different animal models in pre-clinical or clinical studies [142,144,153,154,155]. Studies have been successfully inventing electroporation for therapeutic gene transfer to treat ALI/Acute Respiratory Distress Syndrome (ARDS) in a porcine model [156], and pulmonary fibrosis [157]. In the PH context, electroporation was utilized in a few studies. To investigate the role of the G-protein coupled receptor CXCR4 in the development of PH and vascular remodeling, rats were transplanted with the bone marrow cells electroporated with CXCR4 shRNA. The authors demonstrated that CXCR4 specific inhibition in bone marrow cells reduced PH and vascular remodeling via decreasing bone marrow‑derived cell recruitment to the lung upon hypoxia [158]. To investigate the role of MCP-1 gene therapy in MCT-induced PH, several different protocols of intramuscular gene therapy were implemented. Specifically, intramuscularly injection with the simple naked DNA encoding a 7-NH_2_terminus-deleted dominant negative inhibitor of the MCP‑1 gene (7ND MCP‑1) was compared with the injection of 7ND MCP‑1 achieved by electronic pulses. Interestingly, a significant 7ND MCP-1 protein in plasma was detected only in the group of rats, which underwent electroporation. Both approaches of MCP‑1 gene transfer significantly inhibited the progression of MCT‑induced PH, as evaluated by decrease of right ventricular systolic pressure (RVSP), right ventricular (RV) hypertrophy, and mononuclear cell infiltration into the lung [159]. Despite all mentioned obstacles, in vivo electroporation of the lung is still an attractive method with potential optimization for gene therapy, which also provides enormous benefits for the treatment of many pulmonary infectious diseases [160,161].

### 2.2. Viral Delivery Systems

Stable transfection involves the use of viral vectors to efficiently deliver NAs and targets a wide variety of cells [162]. Viral delivery systems such as lentiviral, adenoviral, oncoretroviral, and adeno-associated vectors (AAVs) are used for transferring NAs even in hard-to-transfect vascular cells.

#### 2.2.1. Lentiviral Gene Transfer

The process of lentiviral transduction includes first the lentiviral production by the human embryonic kidney (HEK)‑293T (HEK‑293 cells expressing the large T‑antigen of simian virus 40). HEK293T cells initially transfected with plasmids containing the lentiviral packaging, envelope genes, and the target sequence, produce lentiviral particles [163]. The lentivirus packaged with this target sequence is used to transduce primary pulmonary vascular cells. Third generation lentiviruses have various advantages over the second generation as the viral *tat* gene is removed and Tat-independent 5’ long terminal repeat (LTR) is added. The original viral packaging components are eliminated and the remaining are distributed in two plasmids. To prevent the repackaging of integrated genes, a modified 3’ LTR is added, making them ‘self-deactivating’ and the packaging capacity of third-generation lentiviruses is up to 8 kb [163]. Genetic manipulation using third-generation lentiviruses is implicated for transduction of PAECs, PASMCs, and pulmonary arterial fibroblasts (PAFs) in vitro [164,165,166,167,168,169] and in the context of various CVDs in vivo [168,170]. The application of lentiviruses ranges from lentiviral-mediated gene expression, regulation of the immune system, reprogramming of stem cells, and vaccine development [171]. Lentiviruses provide a fast, efficient, economical replacement to genetically modified animals to study disease mechanisms and develop potential therapeutic tools In vivo [172]. Preventive approaches using lentiviral vectors in diseases occurring due to vascular wall remodeling have been used such as aortic aneurysm, atherosclerosis, and restenosis [167,173,174]. In the context of PAH, various research groups have successfully used lentiviral constructs. See Table 1 for the details.

##### Transgelin

Transgelin (*Tagln*) gene codes for one of the earliest markers of smooth muscle differentiation SM22α, which regulates actin cytoskeleton rearrangement, phenotypic modulation of vascular SMCs, SMC proliferation, and migration [175,176,177]. A lentiviral vector was constructed and applied to inhibit *Tagln* gene expression in rat PASMCs in vitro and after intratracheal instillations in rats in vivo. The inhibition of *Tagln* expression attenuated hypoxia increased RVSP, as well as cardiac and pulmonary vessel remodeling [178].

##### Galectin-3 (Gal-3)

Galectin-3 (Gal-3) is a β‑galactoside‑binding protein belonging to the member of the galectin family having a conserved carbohydrate-recognition domain [164]. Gal‑3 is associated with increased risk for incident heart failure and mortality and is a predictive biomarker [179]. Gal‑3 has been implicated in EndoMT, a process, which contributes to the pathogenesis of PAH by accumulating α-smooth muscle actin-expressing mesenchymal-like cells in obstructive pulmonary vascular lesions [180]. Intratracheal application of *Gal-3* knockdown and overexpression lentivirus was used in MCT‑rats to further investigate its role in EndoMT in PAH. Overexpression of Gal-3 exacerbated RVSP and RV hypertrophy while the lentiviral knockdown led to a reduction in the RV hypertrophy [164].

##### Twist1

The transcription factor Twist-related protein 1 (Twist1) is known to play a role in lung vascular permeability and dysfunction, pulmonary fibrosis, pulmonary edema, and lung angiogenesis [181,182,183]. Twist1 implication in PH is also related to its contribution to EndoMT and is upregulated in patients with PAH [132,184]. Lentiviral knockdown of Twist1 using shRNA reduced the hypoxia-induced EndoMT as well as the accumulation of αSMA-positive cells in the fibrin gel implanted on the live mice [185].

##### A Cluster of Differentiation (CD40)

A cluster of differentiation 40 (CD40) is a membrane glycoprotein belonging to the tumor necrosis factor (TNF) superfamily. [186]. CD40 ligand, initially thought to be solely involved in immune modulation, has been implicated in various vascular pathologies and is expressed by many vascular cells including ECs, SMCs, AFs macrophages, and platelets [187,188]. Increased plasma levels of soluble CD40 ligand contributes to the pathogenesis of PAH by upregulating inflammatory chemokines [188]. Tail vein injection of lentivirus vector shRNA-CD40 encoded endothelial progenitor cells led to the amelioration of hemodynamics and reversed vascular remodeling in the MCT-rat model of PAH [189].

##### Hypoxia-Inducible Factor (HIF)

Hypoxia-inducible factors 1 and 2 are master transcription factors that are controlled by their respective alpha subunits by the cellular levels of oxygen [190]. Plentiful reports designate that both HIF‑1s critically contribute to pulmonary vascular remodeling and PH [54,191,192,193], with HIF‑1α more essential for SMCs, while HIF‑2α rather specific to ECs of pulmonary vasculature [54,194,195]. Lentivirus-mediated delivery of HIF‑1α shRNA by intratracheal instillation before exposure to hypoxia on the manifestations of hypoxia-induced PH was assessed. The successful delivery of HIF‑1α shRNA into the pulmonary arteries effectively suppressed hypoxia-induced upregulation of HIF‑1α, accompanied by the prominent attenuation of the symptoms associated with hypoxia‑induced PH, including the elevation of pulmonary arterial pressure (PAP), RV hypertrophy, and hyperplasia of PASMCs, as well as the muscularization of pulmonary arterioles [196]. Intraperitoneal injection of HIF‑2α antisense oligonucleotides reduced vessel muscularization, an increase of pulmonary artery pressures, and RV hypertrophy in mice exposed to hypoxia [195].

#### 2.2.2. Adenoviral Gene Transfer

Another way of successful transfection of difficult to transfect pulmonary vascular cells is the usage of replication-deficient recombinant adenoviruses. Nowadays, adenovirus vectors provide a solid platform for efficient gene transfer for in vitro, ex vivo, and in vivo gene transfer [197,198]. The most common adenovirus (Ad), used in gene transfer in human C adenovirus serotype 5 (Ad5), given the ability to infect a wide group of different cell types and the capacity to harbor large genes in the genome [199]. The intracellular delivery of Ad5 occurs by the receptor-mediated endocytosis pathway [197], via sequential attachment through coxsackievirus B-adenovirus receptor (CAR) [200], internalization (alphavbeta3 and alphavbeta5), and penetration (alphavbeta5) of the endosomal membrane [201,202]. Furthermore, the contribution of alternate receptors, as heparan sulfate glycosaminoglycans and MHC class I alpha2 domain also reported [203,204]. One of the major hindrances of the efficient Ad5-transduction is the cell-surface expression of its primary attachment receptor CAR [205]. Various strategic approaches are implemented to enhance the entry of Ad5 into CAR-deficient cells, including genetic capsid modification, pseudotyping Ad5 vectors with both the fiber and penton from other serotypes, employment of bispecific adapters or peptides, and chemical modifications [205]. ECs have been observed to be relatively refractory to Ad5 infection due to the low CAR expression [206,207]. Similarly, SMCs of various origins reveal modest expression of the CAR receptor [208]. The genetic capsid modification of Ad5 with the incorporation of human adenovirus serotype 3 knob domain (Ad5/3Luc1) enhanced the infectivity of hPAECs in vitro, providing a 26-fold increase compared to Ad5 [209]. To specifically target the Ad5 to pulmonary endothelium, a bispecific mAb9B9 antibody-anti-knob conjugate, which associates with the fiber protein in the Ad capsid and the pulmonary endothelial marker angiotensin-converting enzyme (ACE) has been generated. These Ad vectors, selectively targeting Tryptophan hydroxylase-1 (*Tph1*) in pulmonary endothelium were successfully used in vivo in chronic hypoxia-induced PAH in rats [210]. To bypass CAR-dependent infection of human vascular SMCs, transduction experiments using chimeric CD46-utilising Ad5/Ad35 vectors, comprising the Ad5 capsid pseudotyped with the Ad35 penton (Ad5/F35/P35), significantly facilitated transduction efficiency [211]. Numerous lines of evidence point to the successful use of adenoviral-mediated delivery in preclinical rodent models of PH [197,198,199,200,201,202,203,205,206,209,210,211,212,213,214].

##### Peroxisome Proliferator-Activated Receptor Gamma (PPARγ)

Transduction of human PASMCs with adenovirus encoding PPARγ demonstrated to significantly increase PPARγ ligand-dependent expression of Programmed Cell Death 4 (PDCD4) in a concentration-dependent fashion, suggesting that PPARγ confers growth-inhibitory signals in hypoxia exposed human PASMCs through PDCD4 [212]. Furthermore, adenoviral-mediated overexpression of constitutively active PPARγ impaired the activation for 5‑hydroxytryptamine (5‑HT) signaling, implicated in vascular contraction, and remodeling in PAH [215].

##### Calcitonin Gene-Related Peptide (CGRP)

Calcitonin gene‑related peptide (CGRP), produced by alternative splicing of the Calcitonin gene, is one of the most potent and effective vasodilators, which has been shown to regulate pulmonary circulation under pathophysiological conditions and [216,217,218]. CGRP is an effective treatment both for human PH and in rodent models of PH [219,220,221]. Overexpression with adenovirus encoding for pre-pro-Calcitonin gene-related peptide (CGRP) demonstrated to inhibit the proliferation of both aortic SMCs and PASMCs in vitro, suggesting that CGRP may contribute to the pulmonary vascular response to chronic hypoxia in vivo [213]. This assumption was later confirmed by studies in which mice after intratracheal administration of AdRSVCGRP exhibited attenuated pulmonary arterial pressure (PAP), pulmonary vascular resistance, RV hypertrophy, and pulmonary vascular remodeling [222,223].

##### Nitric Oxide (NO)

Nitric oxide (NO) an endogenously synthesized, diffusible gas, with potent cardiovascular protective effects produced by a group of nitric oxide synthases (NOS). As a pulmonary vasodilator, NO plays a key role in the regulation of pulmonary vascular tone, modulating vascular smooth muscle contraction, impeding platelet aggregation, and inhibiting vascular SMC proliferation and migration [224,225,226,227,228,229]. Impaired bioavailability of endothelium-derived NO, endothelial dysfunction, reduced expression and/or activity of key regulators of the pulmonary circulation tone (endothelial NO synthase (eNOS) and inducible NO synthase (iNOS)), all contribute to the pathogenesis/pathophysiology of PAH [229]. Endothelial NOS is the predominant source of NO production in the pulmonary circulation [230,231]. Constitutive eNOS gene transfer (AdCMVceNOS) to balloon-injured rat carotid arteries restored vascular NO production and reduced neointima formation [232], while overproduction of eNOS-derived NO inhibited RVSP, pulmonary vasculature remodeling, and RV hypertrophy in chronic-hypoxia induced mice [233]. Aerosolized recombinant adenovirus containing the constitutive eNOS gene (AdCMVceNOS) augmented ceNOS expression and activity, and reduced acute hypoxic pulmonary vasoconstriction in rat lungs [234]. Similarly, aerosol delivery of the iNOS gene augmented pulmonary NO production and significantly reduced hypoxic pulmonary hypertension and pulmonary vascular remodeling in rats [235].

##### Kv1.5

The oxygen-sensitive, voltage‑gated potassium channels (Kv) function by regulation of membrane potential and vascular tone [236]. Kv1.5, encoded by the *KCNA5* gene, is an important hypoxia‑sensitive Kv channel α subunit, that forms functional homo‑ and heterotetrameric Kv channels in PASMCs. Kv1.5 contributes to the pathogenesis of PH by causing sustained depolarization, which increases intracellular calcium and K+, thus regulating cell proliferation and apoptosis [236,237]. Acute hypoxia selectively reduced Kv1.5 channel activity in PASMCs [238]. Overexpression of rat PASMCs with the human *KCNA5* gene using Lipofectamine accelerates apoptotic volume decrease, an increase of Caspase-3 activity, and apoptosis induction [110]. Orotracheally nebulized Ad5 carrying human Kv1.5 restored the O_2_-sensitive K+ current of PASMCs, normalized hypoxic pulmonary vasoconstriction, diminished pulmonary vascular resistance, but improved cardiac output, which was linked with regression of RV hypertrophy and PA medial hypertrophy [239].

##### Forkhead Box O (FOXO)

The Forkhead box O (FoxO) subfamily of FOX family evolutionary conserved transcription factors are critical for the regulation of cell cycle arrest and induction of apoptosis [240,241,242]. Experiments utilizing targeted depletion of FoxO1 specifically in SMCs point toward a causal SMC‑specific role of FoxO1 in PH development [55]. The transduction with a non-phosphorylatable, constitutively active adenovirus carrying FoxO1 affected proliferation and apoptosis of both control human PASMCs and rat PAH PASMCs, signifying that constitutive activation of FoxO1 inhibits the hyperproliferative and apoptosis-resistant phenotype of PASMCs in vitro [55]. Selective delivery of Ad-Foxo1 to the lungs of MCT‑treated rats resulted in a reduction of the right ventricular systolic pressure (RVSP) and improvement of pulmonary vascular remodeling, which corresponded to suppressed cell proliferation and enhanced apoptosis in pulmonary arteries [55].

##### Vascular Endothelial Growth Factor (VEGF)

Another example of the positive adenovirus-mediated gene delivery came from the studies employing the transfer of the vector carrying the pro-angiogenic vascular endothelial growth factor (VEGF). The prime hypoxia-inducible factor, VEGF belongs to the VEGF family, which comprises several members: VEGF (or VEGF-A), VEGF-B, VEGF-C, and VEGF‑D, VEGF‑F, placental growth factor (PlGF) ligands, and their receptors VEGFR-1,‑2 and ‑3 [243]. Initially described as a permeability factor of the endothelium, VEGF presently recognized as a potent angiogenic factor, inducer of proliferation, sprouting, migration, and tube formation of ECs, concomitantly to its pro-survival role in ECs [244,245,246,247]. Adenoviral-mediated VEGF delivery by intratracheal instillation protected rat lungs exposed to chronic hypoxia against PH development, as compared with rats pretreated with the control vector [248].

##### Angiostatin

Generated by proteolysis of plasminogen, Angiostatin is a potent and specific naturally occurring inhibitor of angiogenesis [249]. Angiostatin inhibits the proliferation and migration of ECs [250,251]. The mice after intratracheal administration of adenovirus vector expressing secretable angiostatin K3 (Ad.K3) showed more severe PH, assessed by higher RVSP and worse pulmonary vascular remodeling, indicating that inhibition of hypoxia-induced angiogenesis exacerbates PH development [252].

##### BMPR2

The *BMPR2* gene, a member of the transforming growth factor superfamily of receptors, was identified as the major risk factor for hereditable PAH. More than 450 heterozygous germline mutations have been described in the *BMPR2* gene [253]. Approximately 70% of patients with hereditary PAH and 20% of patients with incident idiopathic PAH are thought to be driven by mutations in the *BMPR2* gene. Multiple lines of evidence showed that BMPR2 presence in pulmonary arteries is a critical determinant of PAH, as both the gene mutations and its low expression are tightly associated with disease pathogenesis, progression, and outcomes [15,49,254,255]. Thus, the rationale to restore BMPR2 expression to treat the PAH appears to be a reasonable gene therapy approach. BMPR2 targeted delivery to pulmonary vascular endothelium in rodent PAH models attenuates hypoxic pulmonary hypertension [256].

#### 2.2.3. Adeno-Associated Viral Gene Transfer

Initially discovered, as a contaminant of adenovirus preparations, the adeno‑associated virus (AAV) of the *Parvoviridae* family became one of the most investigated in gene transfer and gene therapy approaches recently [257]. AAVs have attracted considerable attention due to apparent lack of pathogenicity, low cytotoxicity and immunogenicity, and the ability to infect both dividing and non-dividing cells [258]. The single-stranded genome of AAVs, which is about 4.7 kb in length, comprises the inverted terminal repeats (ITRs). ITRs, which are required for genome replication and packaging, are two flanking open-reading frames encoding for three genes Rep (Replication), Cap (Capsid), and aap (Assembly). The Rep gene encodes proteins, which are required for viral genome replication and packaging, while the Cap gene encodes for structural capsid proteins, which contribute to cell binding and internalization [259,260]. To date, 13 AAV serotypes and over 100 AAV variants were identified from adenovirus stocks or human/nonhuman primate tissues [261,262]. The differential cell or tissue tropism of various AAV serotypes is primarily determined by interaction with specific receptors [262], although the exact mechanism of tissue‑specific tropism of many distinct AAV serotypes remains largely unknown. The most predominant strain found in humans, the AAV serotype 2 is the best‑characterized AAV so far. An establishment of the first successful infectious clone of AAV2 [263] served as a foundation of the generation of recombinant AAV2 (rAAV2) vectors, which concomitantly to the absence of pathogenicity, reveal a wide range of infectivity, and the ability to establish long-term transgene expression in mammalian cells [264]. Although rAAV2 vectors have been shown to transduce rat aortic vascular SMCs [265] and HUVECs in a time and dose-dependent manner in vitro [266], their use provided conflicting results in vivo [265,267,268]. In comparative studies, rAVV1 and rAAV5 were reported to have higher tropism for ECs and SMCs over rAAV2 both in vitro and in vivo [267,269,270]. To improve specific delivery of AAVs into the desired location various strategies, as the mixing of capsid subunits of different AAV serotypes, insertion of targeting peptides, or other ligands into the structural context of the AAV capsid are currently implemented. To increase the delivery of AAVs into vascular SMCs and ECs, the chimeric AAV capsid variant AAV2.5, which combines the enhanced muscle transduction efficiency of AAV1 and maintained receptor binding of AAV2 was developed by rational design strategy approach [271,272]. Transfection of human coronary artery vascular SMCs with AAV2.5 demonstrated the highest efficiency in terms of in vitro transduction, while in vivo AAV2.5 resulted in effective and long-term transduction of vascular SMCs [273]. Recently, a novel strategy was reported where vectors with true specificity for a target tissue are selected by screening for AAV to display peptide libraries under circulation conditions in a murine model. In this report, the authors used in vivo screening and next-generation sequencing to select an AAV capsid showing extraordinary efficient endothelium-specific transduction [274].

##### Angiopoietin-1 (Ang‑1)/Tie2

The development of vascular structures occurs under the control of a muscle‑secreted ligand Angiopoietin‑1 (Ang1). In addition to its critical role in the vasculogenesis process, Angiopoietin 1 (Ang‑1) via its endothelium-specific tyrosine kinase‑2 (Tie2) receptor also controls both physiological and pathological angiogenesis [275,276]. In PAH, the Ang1/Tie2 pathway is upregulated, increasing the endothelium-derived growth factors synthesis, further contributing to PASMC hyperplasia [277,278]. Blocking of the interaction between Ang1 ligand and Tie2 receptor in the lungs prevented PAH after pulmonary arterial injection of AAV containing an extracellular fragment of the Tie2 receptor (AAV-sTie2) both in MCT‑challenged rats and in rats injected with AAV‑Ang‑1 [279]. Interestingly, cell-based gene transfer of Ang-1 to the pulmonary microvasculature of MCT-treated rats provided opposing results, proposing that the Ang-1/Tie-2 axis protects pulmonary endothelium against MCT mediated injury [280].

##### Prostacyclin Synthase (PGIS)

Pulmonary vasoconstriction with a reduced vasodilatory response is one of the prominent features classically associated with PAH [281]. Prostacyclin synthase (PGIS), a P450 enzyme, is involved in the synthesis of prostacyclin which is a vital cardioprotective hormone released by ECs in the pulmonary circulation [282]. Reduced expression of PGIS enzyme has been found in the lungs of patients with severe PH [283] and engineered cell therapy to deliver PGIS has shown a great promise by attenuating PAH and cardiovascular remodeling in MCT experimental model of PAH [284]. Intramuscular injection of either AAV serotype 1 or AAV serotype 2 of human PGIS significantly inhibited the increase in RVSP and attenuated both pulmonary vascular and cardiac remodeling of mice after chronic hypoxia [285,286] and rats after MCT-treatment [287]. Noninvasive luminal airway lung delivery of AAV5- and AAV9CBhPGIS gene transfer vectors significantly attenuated the severity of MCT-induced PAH and prevented cardiac and pulmonary vascular remodeling [288].

##### Interleukin 10 (IL‑10)

Interleukin 10 (IL‑10) is a multifunctional cytokine with numerous pleiotropic effects in immunoregulation and inflammation. Produced by Type‑2 helper (Th1) lymphocytes IL-10 downregulates the expression of cytokines in Th1 lymphocytes and macrophages [289]. IL‑10 inhibits vascular SMC activation and proliferation both in vitro and in vivo [290,291,292]. An intramuscular injection of AAV5-IL‑10 into apolipoprotein E (ApoE)‑deficient mice obstructed atherogenesis [293], while intramuscular injection of AAV1‑IL‑10 resulted in a significant reduction of neointimal proliferation and inhibition of inflammatory cell infiltration in aortic allografts [291]. An intramuscular injection of AAV1 expressing IL‑10 prevented the development of PAH in the experimental rat MCT model [292]. IL‑10 transduction of MCT-treated rats improved the survival of the PAH rats, reduced the PAP, RV hypertrophy, and medial wall thickness of the pulmonary artery, which was associated with reduced macrophage infiltration and vascular cell proliferation [292].

##### Sarco-/Endoplasmic Reticulum Calcium‑ATPase (SERCA)

The sarco‑/endoplasmic reticulum calcium‑ATPase (SERCA), a transporter that regulates intracellular calcium dynamics [294]. A failure in SERCA regulation contributes to augmented cytosolic calcium concentration, which plays a central role in cell death and survival, specifically controlling vascular SMC proliferation and neointima formation [295]. SERCA expression is downregulated in various rodent vascular injury and experimental models of PAH, synthetic SMCs of atherosclerotic vessels, small hypertrophied pulmonary arteries, and human PASMCs of patients with PAH [198,296]. The gene transfer of SERCA2a via intratracheal delivery of AAV1.SERCA2a resulted in a decrease of RVSP, PAP, and attenuation of vascular remodeling, RV hypertrophy, and fibrosis in MCT-treated rats compared with control AAV1/β‑galactosidase administered animals. In the prevention protocol, a single intratracheal injection of aerosolized AAV1.SERCA2a prevented PAH in the MCT‑injected rats [198]. Furthermore, aerosolized AAV1.SERCA2a gene transfer improved myocardial electrophysiological remodeling and ventricular tachyarrhythmia’s susceptibility, indicating the efficacy of SERCA2a non‑cardiac gene therapy approach for arrhythmia suppression [297]. Nebulization of AAV1.SERCA2a, which is a Yukatan miniature swine model of chronic PH, resulted in attenuation of pulmonary vascular resistance, and a trend towards better long-term survival compared to control animals [298].

**Figure 1:**
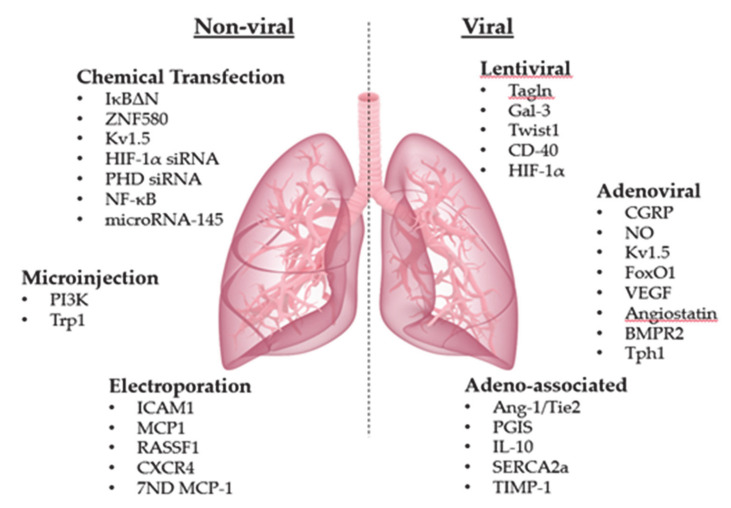
Non-viral and viral delivery systems in pulmonary vascular cells. 7‑NH_2_terminus-deleted dominant negative inhibitor of the MCP-1 (7ND MCP‑1); Angiopoietin 1 (Ang‑1); Angiotensin II (Ang II); Bone morphogenetic protein receptor type 2 (*BMPR2*); Cluster of differentiation 40 (CD‑40); Calcitonin gene-related peptide (CGRP); C‑X‑C chemokine receptor type 4 (CXCR-4); Galectin‑3 (Gal‑3); Fas-induced apoptosis (FIA); Forkhead box O1 (FoxO1); Hypoxia inducible factor‑1α (HIF‑1α); Inhibitor of kappa B mutant (IκBΔN); Intercellular adhesion molecule-1 (ICAM-1); Interleukin 10, (IL‑10); Monocyte chemoattractant protein-1 (MCP‑1); Nitric Oxide (NO); Nuclear factor κB (NF‑κB); Phosphatidylinositol 3-kinase (PI3K); Prolyl hydroxylase domain (PHD); Prostacyclin synthase (PGIS); Ras association domain family 1A (*RASSF1A*); Sarco‑/endoplasmic reticulum calcium-ATPase 2a (SERCA2a); SMC-specific Rho-associated coiled-coil containing protein kinase 2 (*SM22α/ROCK2*); Tissue inhibitor matrix metalloproteinase 1 (TIMP‑1); Transgelin (*Tagln*); Transient receptor potential-1 (Trp1); Tryptophan hydroxylase-1 (*Tph1*); Tyrosine-protein kinase receptor (Tie2); Twist related protein 1 (Twist); Vascular endothelial growth factor (VEGF); Voltage-gated potassium channel member 5 (Kv1.5); Zinc Finger Protein 580 (ZNF580).

A schematic representation of described in vitro and in vivo gene transfer approaches is summarized below (Figure 1).

## 3. Pulmonary Gene Delivery in PAH: Obstacles, Challenges, and Prospective

PAH is a devastating disease, which nowadays represents a growing interest in human gene therapy. The lung is an attractive tissue for gene therapy interventions, as various gene delivery strategies including intranasal, intravenous, intratracheal instillation, or aerosol previously employed in various pulmonary diseases [161]. A large body of evidence has demonstrated that gene therapy approaches in preclinical PAH were successful in the restoration of deficient gene expression and correction of the dysregulated pathway, thereby improving pulmonary vasculature remodeling, inhibiting disease progression, and reversing the established disease [233,299,300,301,302,303]. Local airway delivery to the lungs, including intratracheal instillation or inhalation with or without bronchoscope, demonstrates lower endonuclease activity, minimizes systemic side effects, and avoid liver metabolism and hepatic absorption [304,305,306]. While intratracheal administration of adenoviruses revealed transgene expression in epithelial cells [307,308], the overexpression of eNOS was concomitant with a reduction in acute pulmonary vasoconstriction indicating EC-specific expression [234]. Thus, this mode of administration has proven to affect pulmonary vascular cells in experimental rodent models of PAH [248,252]. For example, intratracheal instillation of the adenovirus TIMP‑1in rats exposed to MCT resulted in attenuation of severe pulmonary vascular remodeling, and a decrease in right ventricular hypertrophy, compared to control‑treated rats [309]. Similarly, an intratracheal aerosol delivery of Kv1.5 gene resulted in an efficient transgene expression in PASMCs, and reduction of hypoxic pulmonary vasoconstriction in rats with established PH [239]. Thus, intratracheal administration via aerosol delivery currently serves as one of the advantageous gene transfer approaches. The intravenous route of administration in experimental rodent models of PH utilized both for viral and non-viral vectors, as a route to deliver gene therapy to the lung via the vasculature. The intravenous route of administration of non-viral vectors into the lung is ascribed with prolonged retention and efficient interactions with the pulmonary vasculature [310,311]. Non-viral transfection vectors might have several advantages, including an almost unlimited packaging capacity, safe application, due to the low immunogenicity, and the ability to package both RNA species and the DNA [312]. However, multiple non-viral vectors are less efficient in gene transfer and confer only transient gene expression in vivo [313], this obstacle could be overcome by repeated administration [314]. The incorporation of tissue‑specific DNA nuclear import signals found in certain promoter sequences or combination with targeting peptide moieties demonstrated to precise and enhance cell targeting in the lung [315,316]. The advent of nanotechnology-based drug delivery tool might serve as a promising gene-therapy approach also in the treatment of PAH. Endothelial dysfunction and elevation of vascular permeability in PAH-lung vasculature, driven by inflammatory responses and hypoxia, causes focal disruptions in endothelial cell basement membranes. Encouragingly, enhanced vascular permeability in PAH contributes to enhanced nanoparticle accumulation in diseased lungs [317,318]. Accumulated in PAH lungs, nanoparticles found to be largely associated with pulmonary vascular ECs and SMCs. Therefore, nanoparticle-based gene therapy approach holds a great therapeutic promise for the treatment of PAH. The main drawback of using virus vectors, in addition to the sophisticated way of their production, their immunogenicity and cytotoxicity. Producing, concentrating, and titrating of adeno‑, lenti‑ and AVV-viral particles, besides that it is a time-consuming and resource-intensive process, demands experience to achieve consistent results. Lentiviruses offer persistent gene transfer in most tissues, though their integration might induce oncogenesis in some applications. Amongst the disadvantage of the lentiviral vectors is the ability of replication, which could lead to incidences of insertional mutagenesis in patients, due to the potential for ongoing viral replication and insertion into the host DNA. Thus, extensive testing for these viruses in vector products, as well as in patients required by many regulatory agencies [319]. Adenoviruses are extremely efficient in the transduction of most cell types and tissues, while their capsid mediates potential inflammatory response. Though both lentiviral and adenoviral vectors serve as efficient vascular gene delivery vectors [320], the transduction of ECs with viral vectors was reported to induce a pro-inflammatory phenotype, with the upregulation of dsRNA-triggered 2′-5′ OA synthetase/RNase L, PKR antiviral, and PI3K/Akt signaling pathways [321,322]. The activation of these pathways reported to the outcome in modifications of surface phenotype and secretion of pro-inflammatory cytokines [323,324]. Studies using organ-cultured pulmonary arteries have demonstrated that adenovirus-mediated gene transfer leads to the induction of host immune responses and apoptosis in vascular ECs, indicating that pulmonary vascular endothelium exhibits a specific antiviral immune activity that affects low gene transfer [325]. In this regard, the generation of helper-dependent adenoviruses, characterized by more persistent gene expression, eliminated capsid-mediated potent inflammatory responses, reveal lower immunogenicity due to the lack of virally encoded genes, and the ability to effectively target airway basal cells in vivo is encouraging [326,327,328]. Although first trials with AAVs vectors demonstrate detrimental immunologic responses and toxicity [329], nowadays AAV-associated gene technology approach is currently among the most frequently used viral vectors systems, which achieved positive results in a number of clinical and preclinical settings [304,330]. AAVs have numerous benefits over the other vectors, which include efficient transduction, long-term transgene expression, and the relative lack of immune responses and toxicities in vivo [331]. Amongst the drawbacks of AAVs is the requirement of helper adenovirus for replication and difficulties in manufacturing of pure AAV stock productions.

Other factors, such as the discovery of new serotypes and the understanding of the tropism of each serotype with the subsequent generation of hybrid serotypes or capsid mutants, can greatly enhance the potential effectiveness of AAV therapy and will improve the efficacy in infecting only the specific cell type of the lung. The major disadvantage of AAV use is the limited transgene capacity of the particles (up to 4.8 kb). Hence, to overcome this limitation, novel dual‑vectors whereby a transgene is split across two separate AAV vectors, have been developed to increase the genome capacity for the AAV vector [332]. All of these trials showed the previously mentioned limitations, inefficiency, and inflammation interactions on cellular levels [142]. Due to the AAV safeties and long-term efficacy, many clinical trials report on successful delivery and transduction of AAVs across various target organs, including the eye, liver, skeletal muscle, and the central nervous system [333]. Thus, we assume that amongst the above-mentioned approaches, the administration with AAV is perhaps the most promising tool that could be safely applied to PAH patients nowadays.

## 4. Conclusions

Gene therapy in PH, though being extensively studied, remains a challenging task. Investigations to transfer non‑viral vectors and adeno or adeno-associated viruses to the lung by tracheal delivery via pulmonary epithelium or by vascular delivery via pulmonary endothelium are being improved to increase the low efficiency and come over the obstacles rising from of the lung-air barrier, lung-blood barrier, inflammatory response and limited duration [334]. Achieving precise delivery of genetic material to the concrete sites of the pulmonary vasculature in the multidimensional structure of the complex lung tissue is another aspect of successful gene therapy. Various innovative approaches such as encapsulation of microRNA-based therapies in nanovectors [335], endothelial progenitor cells and mesenchymal stem cells approach [336], the addition of targeted small molecules in combination with viral vectors [312], the exploitation of gene editing CRISPR-Cas for precise correction of PH-causing mutations are emerging [337]. Future research on gene therapy to develop delivery systems that potentially overcome the resistance in pulmonary vascular cells and lower the cytotoxicity in the context of PH is a need of an hour.

**Table 1:** An overview of different viral gene therapy approaches in pulmonary hypertension. Angiopoietin 1 (Ang‑1); Bone morphogenetic protein receptor type 2 (*BMPR2*); Cluster of differentiation 40 (CD-40); Calcitonin gene-related peptide (CGRP); Galectin-3 (Gal‑3); Forkhead box O1 (*FoxO1*); Hypoxia inducible factor-1α (HIF‑1α); Interleukin 10, (IL‑10); Nitric Oxide (NO); Prostacyclin synthase (PGIS); Sarco‑/endoplasmic reticulum calcium‑ATPase 2a (SERCA2a); Tissue inhibitor matrix metalloproteinase 1 (TIMP‑1); Transgelin (*Tagln*); Tryptophan hydroxylase‑1 (*Tph1*); Tyrosine-protein kinase receptor (Tie2); Twist related protein 1 (Twist); Vascular endothelial growth factor (VEGF); Voltage‑gated potassium channel member 5 (Kv1.5); Mean pulmonary arterial pressure (mPAP); right ventricular systolic pressure (RVSP); ratio of right ventricular weight to left ventricle plus septum (RV/LV+S); wall thickness (WT); pulmonary arterial systolic pressure (PASP); pulmonary arterial diastolic pressure (PADP);% of muscularization of pulmonary arteries (% musc); pulmonary vascular resistance index (PVRI).

	Genes	PH models	Mode of Delivery	Results	References
Lentiviral	*Tagln*	Hypoxia rats	Intratracheal	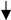 RVSP, 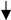 RV/LV+S	[178]
	Gal-3	MCT rats	Intratracheal	OE: 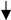 RVSP, 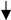 RV/LV+S KD: 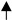 RV/LV+S	[164,180]
	Twist1	Hypoxia mice	Fibrin gel cell delivery	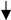 Endo‑MT	[185]
	CD40	MCT rats	Tail vein	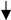 mPAP, 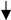 RVP, 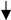 RV/LV+S 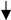 WT	[189]
	HIF-1α	Hypoxia rats	Intratracheal	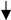 WT, 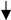 RVSP	[196]
Adenoviral	*Tph1*	Hypoxia rats	Femoral vein	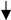 RVSP, 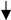 RV/LV+S, 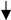 PVR	[210]
	CGRP	Hypoxia mice	Intratracheal	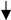 mPAP, 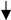 PVR, 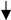 RV/LV+S, 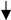 RV/BW	[222,223]
	NO	Hypoxia rats	Intratracheal aerosolization	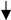 mPAP, 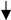 TPRI, 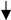 RV/LV+S	[234,235]
	Kv1.5	Hypoxia rats	Intratracheal nebulization	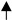 CO, 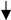 PVR, 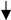 WT, 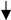 RV/LV+S	[239]
	FoxO1	MCT rats	Orotracheal	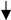 RVSP 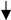 PVRI	[55]
	VEGF	Hypoxia rats	Intratracheal	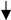 PAP, 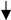 RV/BW, 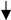 RV/LV+S	[248]
	Angiostatin	Hypoxia mice	Intratracheal	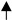 RVSP, 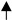 RV/LV+S 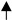 % musc	[252]
	*BMPR2*	Hypoxia rats	Tail vein	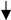 mPAP 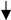 RVSP 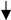 RV/LV+S 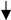 %musc, 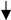 WT	[256]
Adeno-associated viral	Ang-1/Tie2	MCT rats	Jugular veindelivery	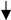 RVSP, 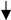 RV/LV, 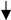 WT	[279]
	PGIS	Hypoxia mice MCT rats	Orotracheal	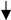 RVSP 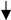 RV/LV+S 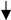 WT	[285,286,287]
	IL-10	MCT-rats	Intramuscular	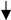 mPAP, 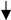 RV/LV+S, 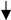 WT	[292]
	SERCA2a	MCT rats, Hypoxia swine	Intratracheal aerosolization Endotracheal nebulization	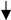 PASP, 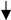 PADP, 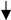 mPAP 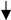 RV/LV+S, 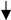 Fibrosis, 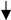 WT, 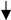 PVRI	[198,297,298]
	TIMP-1	MCT rats	Intratracheal	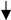 PVR, 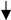 RV/LV	[309]

## Data Availability

Not applicable.
